# Atrial fibrillation ablation using very short duration 50 W ablations and contact force sensing catheters

**DOI:** 10.1007/s10840-018-0322-6

**Published:** 2018-02-19

**Authors:** Roger A. Winkle, Ryan Moskovitz, R. Hardwin Mead, Gregory Engel, Melissa H. Kong, William Fleming, Jonathan Salcedo, Rob A. Patrawala, John H. Tranter, Isaac Shai

**Affiliations:** 1 Silicon Valley Cardiology, Palo Alto Medical Foundation, Sutter Health, E. Palo Alto and Sequoia Hospital, Redwood City, CA USA; 2 Silicon Valley Cardiology, 1950 University Avenue, Suite 160, E, Palo Alto, CA 94303 USA; 3 St. Jude Medical, Inc, St. Paul, MN USA

**Keywords:** Atrial fibrillation, AF ablation, Contact force catheters

## Abstract

**Purpose:**

The optimal radiofrequency (RF) power and lesion duration using contact force (CF) sensing catheters for atrial fibrillation (AF) ablation are unknown. We evaluate 50 W RF power for very short durations using CF sensing catheters during AF ablation.

**Methods:**

We evaluated 51 patients with paroxysmal (*n* = 20) or persistent (*n* = 31) AF undergoing initial RF ablation.

**Results:**

A total of 3961 50 W RF lesions were given (average 77.6 ± 19.1/patient) for an average duration of only 11.2 ± 3.7 s. As CF increased from < 10 to > 40 g, the RF application duration decreased from 13.7 ± 4.4 to 8.6 ± 2.5 s (*p* < 0.0005). Impedance drops occurred in all ablations, and for patients in sinus rhythm, there was loss of pacing capture during RF delivery suggesting lesion creation. Only 3% of the ablation lesions were at < 5 g and 1% at > 40 g of force. As CF increased, the force time integral (FTI) increased from 47 ± 24 to 376 ± 102 gs (*p* < 0.0005) and the lesion index (LSI) increased from 4.10 ± 0.51 to 7.63 ± 0.50 (*p* < 0.0005). Both procedure time (101 ± 19.7 min) and total RF energy time (895 ± 258 s) were very short. For paroxysmal AF, the single procedure freedom from AF was 86% at 1 and 2 years. For persistent AF, it was 83% at 1 year and 72% at 2 years. There were no complications.

**Conclusions:**

Short duration 50 W ablations using CF sensing catheters are safe and result in excellent long-term freedom from AF for both paroxysmal and persistent AF with short procedure times and small amounts of total RF energy delivery.

## Introduction

Radiofrequency (RF) ablation is widely utilized to treat atrial fibrillation (AF). The predominant goal for catheter ablation of AF is durable pulmonary vein (PV) isolation. Virtually, all veins appear isolated at the end of a procedure, but late recurrences can occur and repeat studies often show PV reconnection. Lesion formation for PV isolation and other ablations depend upon RF current delivered, duration of RF energy delivery, and good ablation catheter tissue contact. Increasing the power (and, therefore, current) to 50 W for longer duration lesions results in increased ablation efficacy but is associated with increased complications [1]. Shorter duration 50 W lesions also provide better long-term outcomes but without an increase in complications [2]. The availability of catheters which measure tissue contact force (CF) provides a tool to potentially improve PV isolation and long-term outcomes. Prior studies using CF have used average power of 25–28 W for durations of 40 to 75 s at each site for a total RF energy delivery time of 37.6 to 46.5 min [3–5]. Our study evaluates the feasibility of doing AF ablation using very short duration ablations at a power of 50 W with CF sensing catheter technology.

## Methods

### Patient population

The subjects were consecutive symptomatic patients with paroxysmal or persistent AF undergoing initial AF ablation done by point by point RF ablation with the St. Jude (St. Paul, MN) TactiCath® CF sensing catheter at Sequoia Hospital, Redwood City, California. All patients signed written informed consent. The study was approved by the Western Institutional Review Board. The AF type was categorized as paroxysmal: lasting < 1 week or persistent: lasting > 1 week and < 1 year or requiring pharmacological or electrical cardioversion in < 1 week. Patients with longstanding persistent AF lasting >1 year were excluded.

### Ablation protocol

Our ablation protocol [6] and our periprocedural anticoagulation protocols [7] have been previously described. Antiarrhythmic drugs were stopped at least five half-lives and amiodarone at least 3 months before ablation. The St. Jude EnSite™ Velocity™ system was used in all cases for 3D mapping. All patients underwent circumferential PV isolation and other ablations as clinically indicated. All ablations were at 50 W, including the posterior wall, using the St. Jude TactiCath™ open irrigated-tip CF sensing catheter and the St. Jude Ampere™ RF generator with a target CF of 10–40 g. CF readings of 5–10 g were accepted, provided visual inspection of the CF waveform showed a stable pattern without respiratory variation and was constantly above 0 g. If we did not get adequate CF, the transseptal sheath was exchanged for a St. Jude Agilis™ steerable sheath. We used a 2s ramp time, a catheter irrigation rate of 30 ml/min, and a 50 °C temperature cutoff in the LA and 42° for the RA isthmus. Lower temperature cutoff was used in the RA isthmus to avoid steam pops if the tip was buried in a trabeculation. For patients in sinus rhythm, pacing was undertaken from the distal bipole of the ablation catheter at 10 mA and 2 ms duration during RF energy delivery at a rate approximately 20 bpm faster than sinus. We terminated RF energy delivery several seconds after there was loss of capture, confirmed by sudden return to the sinus rate and loss of atrial capture by the pacing spikes. For patents in AF, we used a target LSI of 5.5–6 at all locations. After the veins were encircled, patients in AF were cardioverted. We evaluated entrance and exit block using a St. Jude Spiral™ circular mapping catheter documenting lack of vein potentials and failure of pacing inside the vein to propagate to the atrium. The esophagus was marked with a thermistor catheter. Our RF lesions were so short that any temperature rise was seen after RF was terminated. If there was a small temperature rise, we did not resume ablation until the esophageal temperature fell back to baseline. After vein isolation, other additional clinically indicated ablations were performed. Isoproterenol was given to look for non-PV triggers and arrhythmia induction performed with bursts of rapid atrial pacing before and during isoproterenol. Non-PV triggers or induced atrial flutters or tachycardias were mapped and ablated.

### Data collection and analysis

For each patient, we recorded preablation age, gender, duration of AF, AF type, prior antiarrhythmic drug therapy, CHADS_2_ and CHA_2_DS_2_-VASC scores, cardioversions, body mass index (BMI), LA size, prior strokes/transient ischemic attacks (TIAs), and the presence of hypertension, diabetes, coronary artery disease (CAD), cardiomyopathy, and obstructive sleep apnea. For each RF energy delivery, we recorded the duration of RF time in seconds, the CF in grams sampled at 50 Hz and averaged over the duration of each RF application, the force time integral (FTI) in gram-seconds (gs), the LSI, and the percent impedance drop during RF energy delivery. FTI is the average CF of each lesion in grams multiplied by the duration of the lesion in seconds, and the LSI was empirically derived in animals to reflect lesion size. The LSI metric is calculated and displayed in real time. LSI is derived using a complex proprietary mathematical formula that takes into account a 6-s moving average of CF and current as well as time. Procedure time was defined as time from groin stick to sheath removal. A successful ablation procedure was defined as no AF, flutter, or tachycardia lasting more than 30 s off of antiarrhythmic drugs after a 3-month blanking period.

### Follow-up

No patients received antiarrhythmic drugs during the blanking period. Patients who went into persistent AF were cardioverted at the end of the blanking period. Patients sent daily transtelephonic ECG strips for 1–3 months after ablation and were seen at 3 months when a 7- to 14-day continuous ECG patch monitor was done. Initial failures were encouraged to undergo a repeat ablation after the blanking period; however, only the initial ablation was used for outcome analysis. Patients were seen or contacted frequently from 3 to 12 months and seen at 1 year when they underwent another 7- to 14-day continuous ECG patch monitor. Thereafter, patients were seen directly or contacted by phone at least annually and arrhythmia records obtained from hospitals and referring physicians. ECG recorders were reissued for arrhythmia symptoms. Pacemaker AF data were utilized when available.

### Statistical analysis

Statistical analysis was done using XLSTAT 2014. Continuous data were described as mean ± standard deviation and counts and percent if categorical. Analysis of variance was done for the FTI, LSI duration of lesions, and average impedance drop during RF energy delivery by ranges of CF. Kaplan-Meier curves were generated for AF-free survival after the initial ablation for patients with paroxysmal and persistent AF and for patients grouped by the percent of the total number of ablation lesions done with CF < 10 g. All statistical tests were two sided, and *p* < 0.05 was considered statistically significant.

## Results

### Patient population

The patient population is summarized in Table 1. There were 31 males and 20 females with an average age of 68.4 ± 9.3 years. Twenty-one patients had paroxysmal AF and 30 patients had persistent AF. Thirteen patients were in AF at the time of ablation. The average duration of AF was 6.1 ± 7.0 years, and patients had failed an average of 0.92 ± 0.91 antiarrhythmic drugs.

**Table 1:** Clinical characteristics

Number of patients	51
Left atrial size (cm)	4.00 ± 0.51 (range 3.0–5.4)
Age (years)	68.4 ± 9.3 (range 46–85)
Body mass index	27.8 ± 5.8 (range 20.9–42.5)
Gender female	20 (39.2%)
Duration of AF (years)	6.1 ± 7.0 (range 0.2–40)
No. of antiarrhythmic drugs failed	0.92 ± 0.91 (range 0–4)
CHADS_2_ score	2.1 ± 0.9
CHA_2_DS_2_-VAS_C_ score	3.2 ± 1.2
Diabetes mellitus	6 (11.8%)
Obstructive sleep apnea	11 (21.6%)
Hypertension	30 (58.8%)
Prior stroke/transient ischemic attack	3 (5.9%)
Prior cardioversion	20 (39.2%)
Coronary artery disease	4 (7.8%)
Dilated cardiomyopathy	5 (9.8%)

### RF energy delivery

**Fig. 1:**
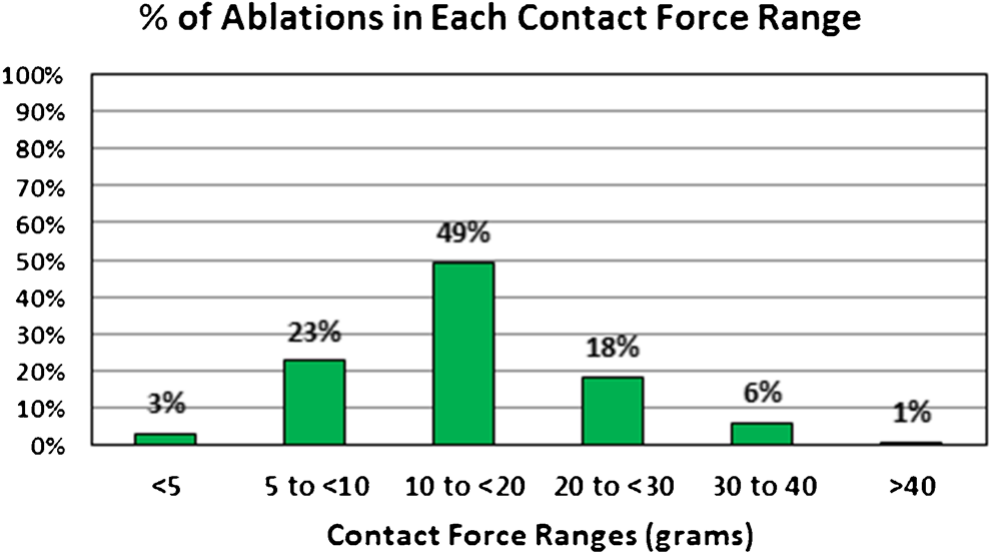
The percentage of the total number of 50 W RF lesions by average CF ranges

**Fig. 2:**
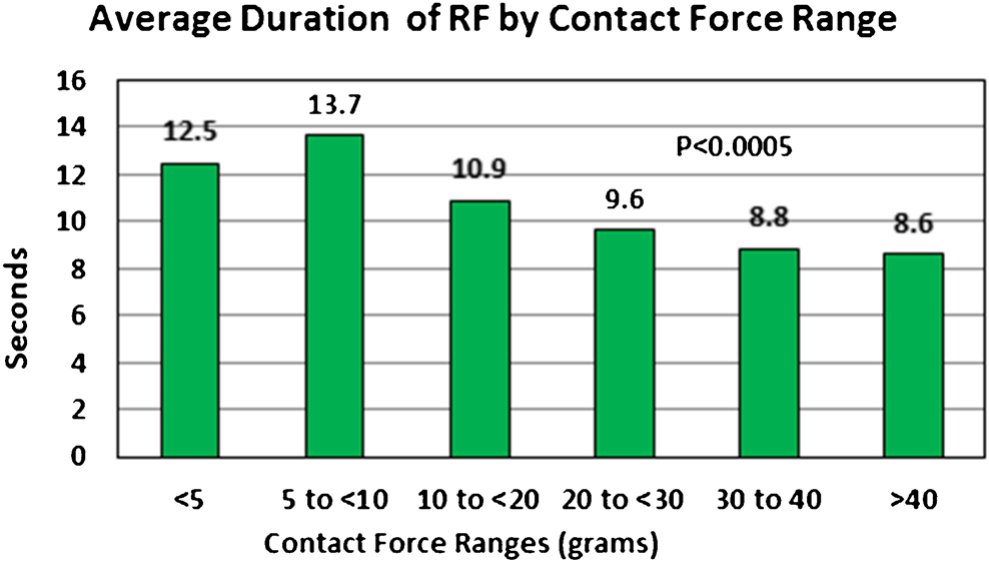
The average duration of 50 W RF lesions by average CF ranges

A total of 3961 RF lesions were delivered in these 51 patients, an average of 77.6 ± 19.1 per patient. All lesions were at 50 W. Figure 1 shows the distribution of lesions by ranges of CF. Only 3% of the lesions were below 5 g of force, and only 1% were > 40 g of force. The largest group was 49% with an average force between 10 and 20 g. The average duration of RF delivery was 11.2 ± 3.7 s. Figure 2 shows the distribution of average lesion duration by CF ranges. For lesions with < 5 g of force, the average duration of RF delivery was 12.5 ± 4.9 s and for CF from 5 to < 10 g, the average duration of each lesion was 13.7 ± 4.4 s. Above 10 g, there was a gradual decline in duration of RF energy delivery down to 8.6 ± 2.0 s for average CF > 40 g (*p* < 0.0005). All PVs showed entrance and exit block at the end of the procedure and failure of pacing around the circumference of the vein to capture. In addition to PV isolation, 15 patients underwent right atrial isthmus flutter ablation for clinical or induced right atrial flutter, 37 patients underwent left atrial roof line ablation, 1 underwent a low posterior line ablation for posterior wall isolation, 3 left atrial flutters were mapped and ablated, and complex fractionated atrial electrograms were ablated in 1 patient. These ancillary ablations were also done at 50 W for short duration, and their RF data is included in the data for each patient. The average procedure time for these patients was 101 ± 19.7 min (range 58 to 180). The total duration of RF energy delivered was 895 ± 250 s (range 307 to 1893).

### LSI and FTI

**Fig. 3:**
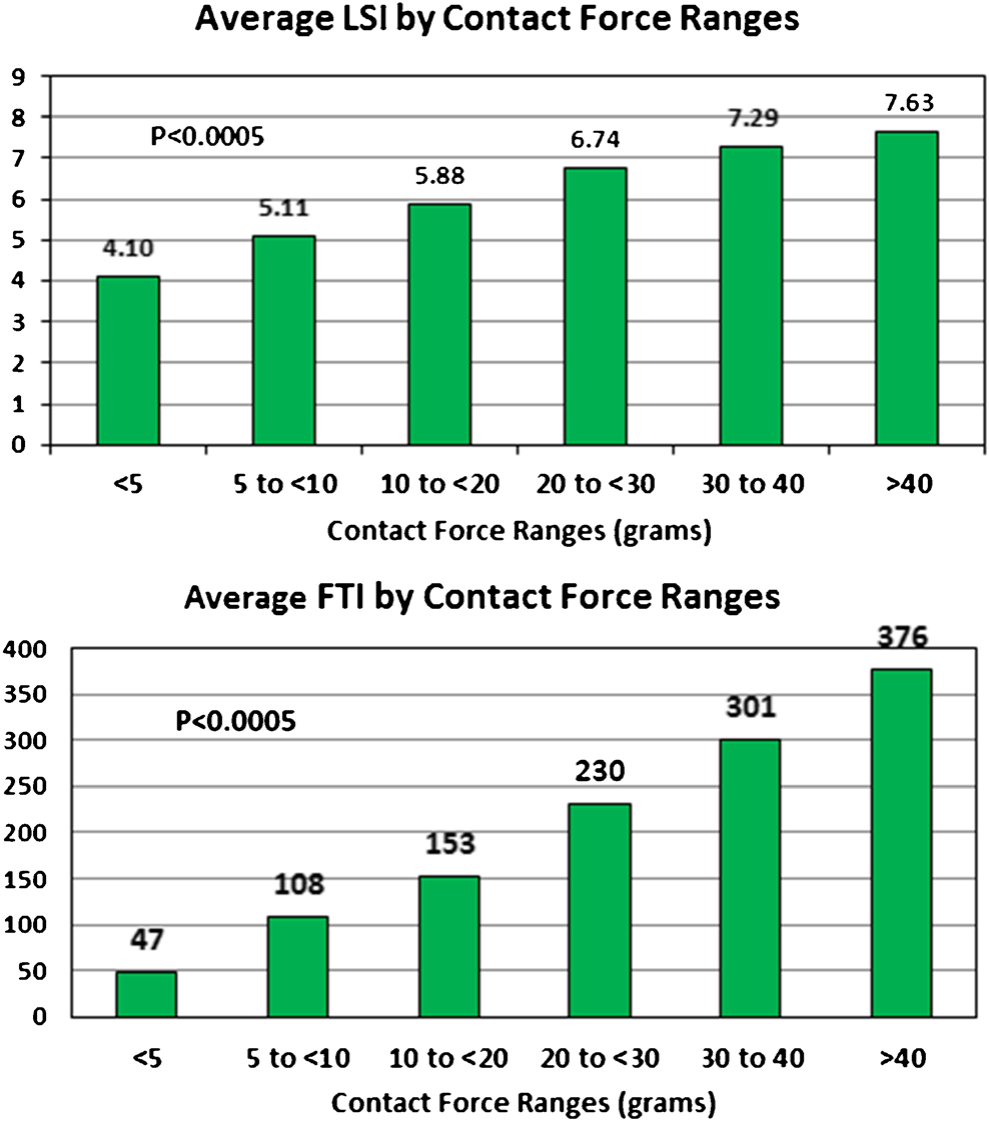
The average lesion index (LSI) (top panel) and the average force time integral (FTI) (bottom panel) by CF ranges

The average LSI for all lesions was 5.9 ± 0.88. Figure 3 (top panel) shows the average LSI by CF ranges. As the CF increased from < 5 to > 40 g, the LSI value increased from 4.10 ± 0.51 to 7.63 ± 0.50 (*p* < 0.0005). Figure 3 (bottom panel) shows the average FTI by CF ranges. As CF increased from < 5 to > 40 g, the average FTI increased from 47 ± 24 to 376 ± 102 gs (*p* < 0.0005).

### Loss of pacing capture and impedance drop during RF energy delivery

**Fig. 4:**
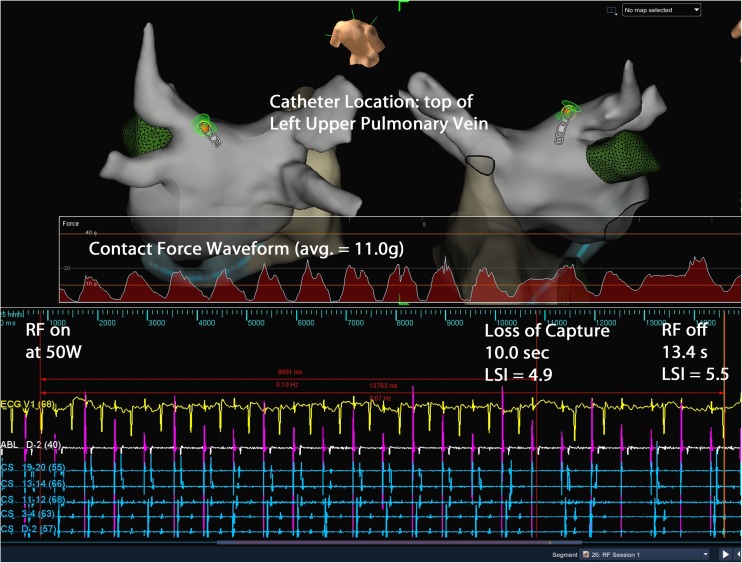
Loss of pacing capture during RF. This figure shows a single lesion created near the top of the left upper pulmonary vein. While pacing the distal bipole of the ablation catheter, RF is turned on at 50 W. After 10 s of RF (when the LSI was 4.9), there is loss of capture. The RF was continued for a total RF time of 13.4 s. The average CF was 11.0 g

**Fig. 5:**
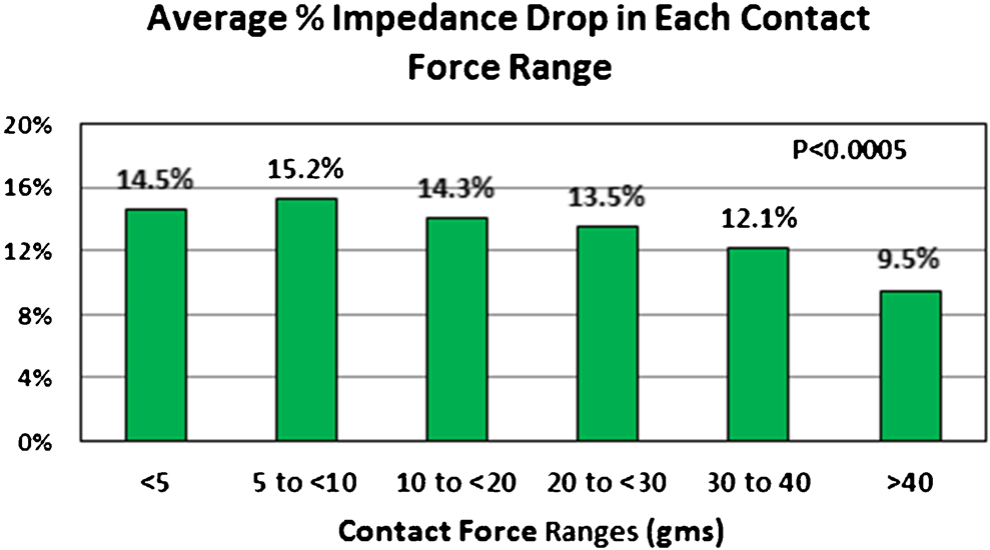
The average percent impedance drop per lesion by CF ranges

To monitor surrogates for lesion formation, we monitored the loss of pacing capture during RF delivery for those patients in sinus rhythm and impedance drops for each RF energy delivery for all patients. Figure 4 shows a typical burn done in sinus rhythm with loss of pacing capture during RF. The average impedance drop for all lesions was 13.5 ± 5.5%. As CF increased, the average impedance drop per lesion decreased from 14.5 ± 5.2% for the 5 to < 10 g force range to 9.6 + 5.2% for CF > 40 g (*p* < 0.0005) (Fig. 5). We did not have an audible or tactile steam pop in any of the 3961 RF applications. No char was noted on any catheters. For all lesions delivered in sinus rhythm, we paced *during* RF energy delivery. Except for an occasional lesion on the anterior portion of the right upper PV, there was always loss of capture prior to termination of RF energy delivery. For lesions on the anterior right upper vein, where there was no loss of capture, we stopped RF at an LSI of 6.0. Rechecking these areas after terminating RF showed no electrograms and no capture after repositioning the catheter. We felt that the proximal pole of the catheter may have been touching tissue and causing anodal stimulation or there may have been adjacent right atrial tissue capture to explain continued atrial capture when the LSI reached 6.0. Prior to terminating the procedure, we paced along all lesion sets to further document failure to capture. All veins were isolated in all patients, and there were no acute reconnections noted.

### Long-term outcomes

**Fig. 6:**
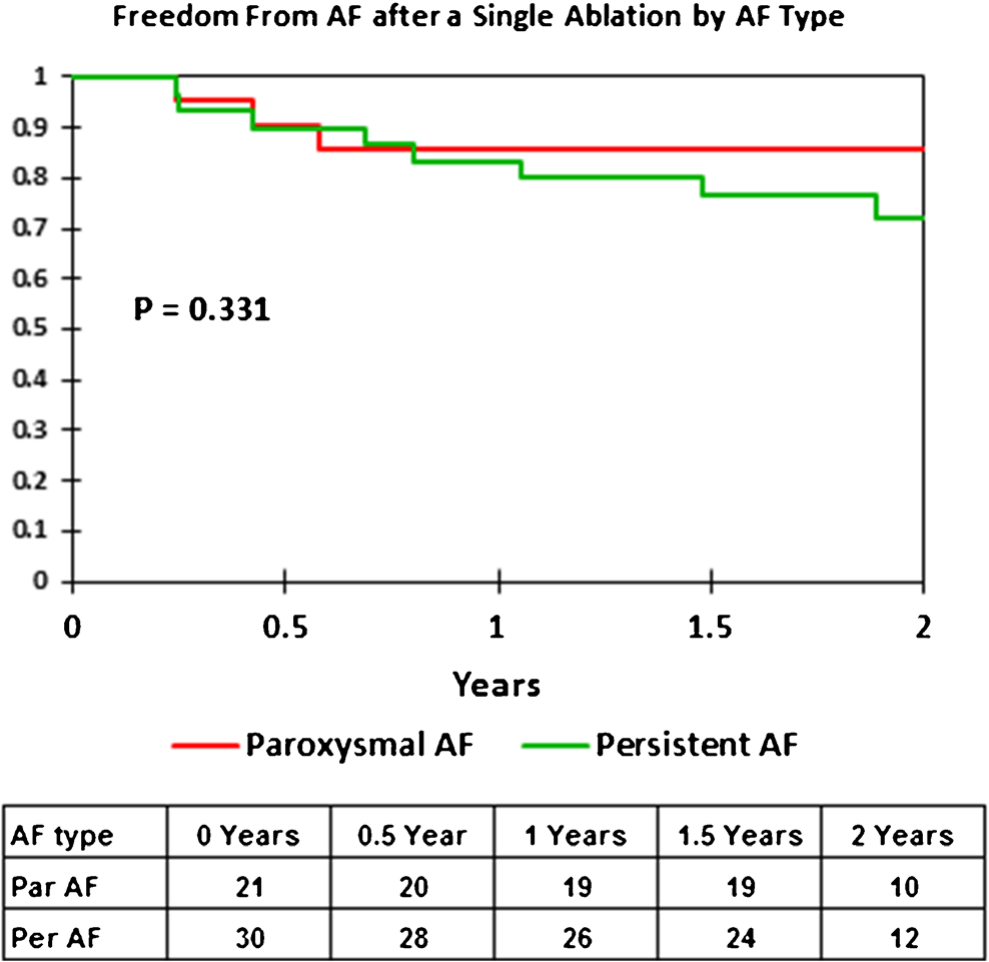
Kaplan-Meier curves showing the single procedure freedom from AF by AF type (red = paroxysmal AF and green = persistent AF)

All patients considered free of AF underwent 3 and 12 month 7- to 14-day continuous monitors. The average follow-up was 1.74 ± 0.61 years. Figure 6 shows the Kaplan-Meier single procedure AF-free rates by AF type (paroxysmal vs. persistent). For paroxysmal AF, the single procedure freedom from AF was 86% at 1 and 2 years. For persistent AF, the single procedure AF-free rate was 83% at 1 year and 72% at 2 years. The difference in long-term outcome between paroxysmal and persistent AF was not significant (*p* = 0.331).

**Fig. 7:**
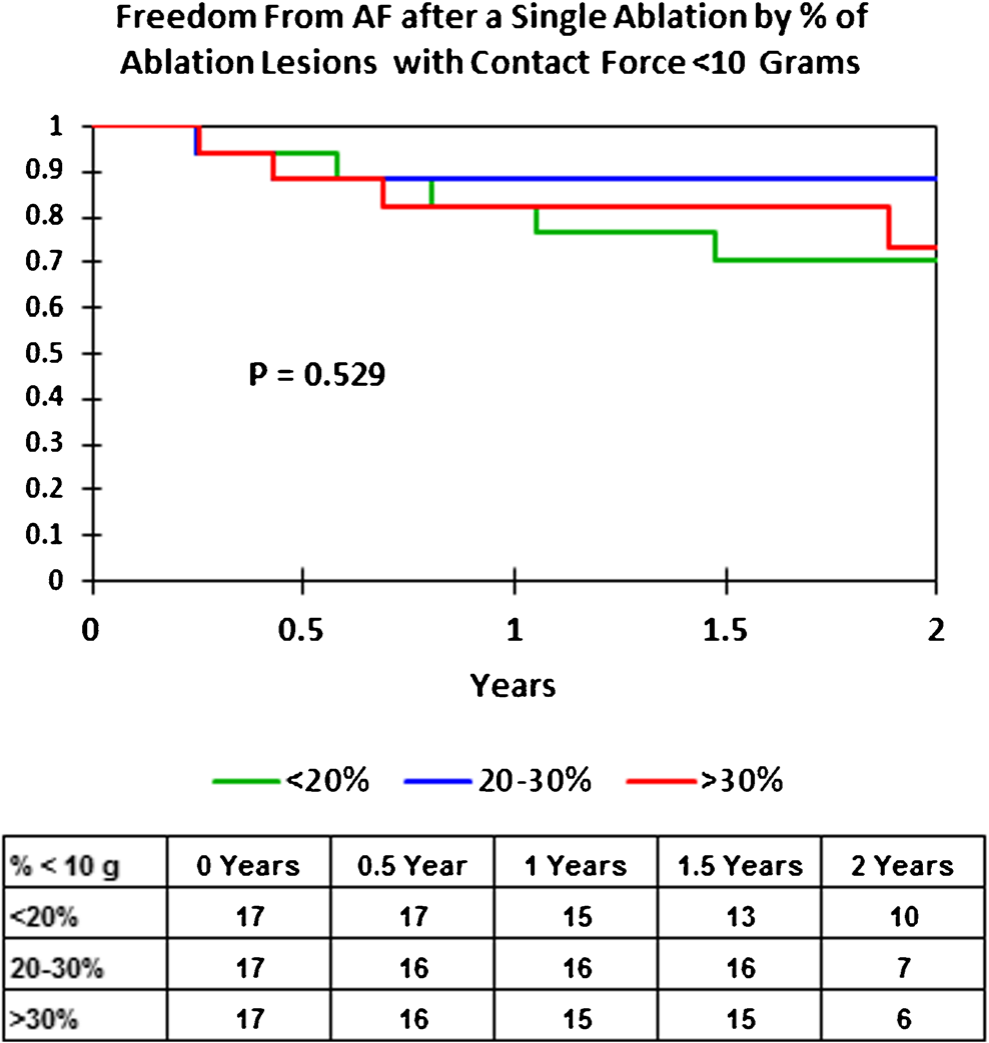
Kaplan-Meier curves showing the single procedure freedom from AF by the percentage of 50 W RF lesions with<10 g of CF in each patient (green = < 20% of the lesions at < 10 g, blue = 20–30% of the lesions < 10 g, and red = > 30% of the lesions < 10 g)

We also examined the single procedure AF-free rate for the entire cohort by the percent of lesions in each patient with average CF < 10 g. We compared patients who had < 20% of their RF lesions at < 10 g to those with 20–30% of the lesions at < 10 g and those with > 30% of the lesions at < 10 g. Figure 7 shows the Kaplan-Meier curves for these three groups. There was no difference in single procedure AF-free rates, suggesting that even the lesions created with < 10 g of force using 50 W, the majority of which were between 5 and 10 g (averaging 8.0 g of force), were making durable lesions. This may have been due to our requiring an optimal CF waveform for CF between 5 and 10 g.

Nine patients returned for a repeat ablation after their initial ablation failed. All had at least one active vein. There was more than one site of vein reconnection of 24.2% and a single site of reconnection in 27.3% of the veins. Eight of the nine patients had atrial arrhythmias eliminated by the redo ablation.

### Complications

There were no instances of stroke or TIA, pericardial tamponade, atrial-esophageal fistulae, PV stenosis, or death.

## Discussion

The main finding of this study is that using CF sensing catheters and 50 W ablations averaging only 11.2 s/burn, we safely isolated the PVs in all patients with much shorter procedure times and total RF energy delivery (< 15 min/patient) than reported in prior studies using lower power and longer duration RF applications [3–5]. We had an excellent single procedure 1 and 2-year freedom from AF.

In the present study, we did point by point ablation to examine the characteristics of each lesion regarding RF duration, loss of pacing capture during RF delivery, impedance drop, and measurement of LSI and FTI. The average duration of all lesions was 11.2 ± 3.7 s. The RF generator we utilized takes 2 s to get up to full 50 W power when RF energy is applied to the catheter. Several animal studies support the use of 50 W ablation for 5–10 s. In an *in vitro* and *in vivo* sheep model, Bhaskaran et al. [8] compared 50 and 60 W ablations for 5 s with the conventional 40 W ablations for 30 s, all delivered at a CF of 10 g. They delivered energy after the RF generator had reached full power. Their 5-s ablation would be comparable to a 7-s ablation in our study. They demonstrated that 50 and 60 W ablations for 5 s achieved transmural lesions and were safer than the 40 W 30-s ablations. Steam pops occurred in 8% of the lesions using 40 W for 30 s and in none of the 5-s 50 and 60 W ablations. When they evaluated 80 W for 5 s, there was an 11% occurrence of steam pops, suggesting an upper limit on safe power for short duration lesions. Another study by Goyal et al. [9] in fresh killed porcine ventricles showed that for 20 g of CF, the time needed to create a 4-mm deep lesion decreased from just over 20 s for 20 W to 6–7 s for 50 W. That study suggested that these high power and short durations might help to reduce collateral injury.

Several previous clinical studies evaluated 50 W ablations using non-CF catheters. Kanj et al. [1] compared 35 vs. 50 W ablations. They randomized 180 patients (85% with paroxysmal AF) to ablation using either an 8-mm non-irrigated catheter or an open irrigated tip catheter (OITC) at either 35 or at 50 W. For the OITC, they showed a 6-month freedom from AF of 82% at 50 W and only 66% at 35 W with shorter fluoroscopy and left atrial times with 50 W. They did note more steam pops, pericardial effusions, and gastrointestinal complaints at 50 W, probably because they ablated at each site for prolonged periods of time and did not shorten the RF delivery time for the 50 W lesions. Bunch and Day [10] reported on the use of 50 W and a “painting” technique where they moved the catheter back and forth across a small area until it was devoid of electrograms and reported no esophageal injuries and an 85% freedom from AF after one or two ablations with a mean follow-up of 338 days. In a previous study, we reported a technique like that of Bunch and Day, termed “perpetual motion,” using open irrigated tip catheters at 50 W [2] for short durations at each site. Compared with lower power ablations for longer durations, the short 50 W ablations had better long-term freedom from AF and shorter procedural, left atrial and fluoroscopy times. There was no increase in complications with the use of 50 W for short durations. The present study extrapolates those observations about short duration 50 W ablations to the use of CF sensing catheters.

The appropriate range of CF and appropriate method for real-time monitoring of CF ablations is still being determined. In the TOCATTA study [4], using 15 to 40 W of power, there was a higher 1-year success rate when the CF was maintained above 20 g. That study also suggested the FTI should be > 500 gs and possibly > 1000 gs for best outcomes. The EFFICAS I study [3], using a median of 25 W of power for up to 60 s at each site, showed fewer PV reconnections when the FTI was > 400 gs. The FTI has been commonly used as a real-time surrogate for durable lesion formation. There are at least four determinants of lesion formation at each site: CF, RF energy duration, RF current, and catheter stability. The only components the FTI considers are the CF and the RF lesion duration. The FTI does not consider either current or power level or catheter stability. Das et al. [11] recently reported on the use of the “ablation index” which is similar to the FTI but also includes power in a weighted formula. They found the ablation index to be superior to FTI for acutely monitoring PV isolation. In our study, we followed the LSI measurement which also utilizes CF, RF duration, and RF current (which reflects delivered power) in determining a real-time number to guide ablations. We also visually incorporated the CF waveform as a measure of catheter stability, especially for ablations done at < 10 g of force. We monitored both impedance drop at all sites and loss of capture for the patients in sinus rhythm and always saw loss of capture and a fall in impedance, indicating good lesion formation by the time the LSI reached a value of 5.5–6.0. This would suggest that when using 50 W ablations for patients in AF, where loss of pacing capture cannot be monitored, one should deliver energy at all sites long enough to achieve an LSI in the 5.5–6.0 range. For patients in sinus rhythm, when ablating the anterior portion of the right upper PV while pacing, one should stop the ablation when the LSI reaches 6.0, even if there is still atrial capture, as the capture may anodal form the proximal pacing electrode of the ablation catheter. Since force quality is not incorporated into the LSI measurement, for CFs between 5 and 10 g, one should carefully monitor CF quality by continuous visual inspection of the CF waveform. If this waveform frequently drops to 0 g or is labile, one should abort that lesion and start anew with higher or more stable CF. When using 50 W ablations, one cannot use the prior target FTI numbers reported in the literature, as these were all derived using RF energy well below 50 W. In our study using short duration 50 W ablations, we did not achieve an average FTI of 400 gs, even for lesions with a CF > 40 g. There was an eightfold range with wide standard deviations (from 47 ± 24 to 376 ± 102 gs) for average FTIs as CF went from < 5 to > 40 g and less than a twofold range with smaller standard deviations (from 4.10 ± 0.51 to 7.63 ± 0.50) for average LSI over the same CF ranges. This suggests that the LSI is a better number to monitor than the FTI, as it takes RF current into consideration and is less variable from lesion to lesion over a range of CFs.

Our study suggests that, when using CF sensing catheters at 50 W, if one wants to use drag lesions, perpetual motion or painting techniques to keep the RF generator on while moving the ablation catheter to a new site, one should remain at each spot for approximately 8–9 s for higher CF sites and for a few seconds longer at lower CF sites, before moving on to the next spot.

Studies have indicated that a fall in impedance during RF energy delivery [12] or loss of pace capture [13] indicates lesion formation. Studies evaluating CF suggest that there is a greater fall in impedance with more CF [14]. For unclear reasons, we found a smaller decrease in impedance as CF increased. This may be due to the fact that we used endpoints of lesion formation (loss of pace capture and LSI) to guide the termination of RF rather than measuring impedance drop at a fixed time after the onset of RF delivery and that our ablation times were very short.

In the TOCCASTAR study [5] for paroxysmal AF ablation, there was no difference in the 1-year freedom from AF when the CF sensing catheter was compared to the non-CF sensing catheter. However, when the data were analyzed comparing patients who received ≥ 90% of the ablation lesions with ≥ 10 g of force to those who had < 90% of their lesions at ≥ 10 g, the group with a higher percentage of high CF lesions achieved a 75.9% 1-year freedom from AF compared to 58.1% for the group where CF was less optimal. In our present study, having a higher percentage of ablation sites at less than 10 g of force did not significantly impact the outcome. In addition, for paroxysmal AF, our 1-year 86% freedom from AF was considerably higher than the overall 67.8% seen in the TOCCASTAR trial for paroxysmal AF. This could be due to our requirement that for CF between 5 and 10 g, the CF waveform must be ideal and consistent with good contact or it could be that 50 W ablations overcome the limitations of slightly lower CF.

Radiofrequency energy delivery to tissue is a complex interaction and is well summarized in a recent review [12]. There is a resistive component adjacent to the catheter electrode which results in local heating and dissipation of radiofrequency energy as heat. This resistive heating depends upon current delivered to the tissue and the resistance seen by the RF generator. Resistive heating probably occurs relatively early in the RF application. Greater resistive heating can be achieved by the use of higher RF power or lower resistance, achieved by the use of larger tip catheters or extra skin electrodes. There is also a secondary passive heating of deeper tissue which increases with longer duration RF applications. Tissue needs to be heated to 50 °C or higher for several seconds to achieve irreversible coagulation necrosis which results in an electrically silent scar. The use of catheter tip cooling and greater energy both result in larger and deeper scar formation. Open irrigated tip catheters, such as the one used in the present study, results in tip temperature increases of only a few degrees Celsius which are associated with 20–30° of tissue temperature increases [15]. Thus, with the open-irrigated tip catheters, one can heat and ablate adjacent tissue at 50 W of power without having catheter tip coagulation in the blood pool. The shorter, higher energy RF applications we used in the present study may also result in fewer complications by achieving rapid local resistive tissue ablation and avoiding deeper collateral passive heating seen with longer and lower power RF applications. Although the present study is too small to examine the rate of infrequent complications, over the past 12 years, we have performed more than 4500 AF ablations using 50 W for short durations, including the posterior wall, with only a single non-fatal atrioesophageal fistula, a single patient with PV stenosis requiring intervention, a pericardial tamponade rate of 0.34%, and a 48-h stroke rate of 0.145%.

## Limitations

This was a single center study. There were a relatively small number of patients evaluated. However, there were almost 4000 ablation applications evaluated and almost five million individual data points examined with 50 Hz sampling. Despite the small number of patients, the excellent long-term single procedure outcomes support the animal data referenced previously and provides “proof of concept” for using this technique in future trials of AF ablation. Although we saw no complications, the number of patients is too small to evaluate for infrequent serious complications that can occur with AF ablation.

## Conclusions

Monitoring the quality of the CF waveform, documenting loss of capture during pacing during RF delivery for patients in sinus rhythm, and following a parameter that takes current or power into account provide a safe and rational method for monitoring short duration RF ablations at 50 W using CF measuring catheters. The use of short duration 50 W RF lesions permits shorter procedures with minimal total RF energy delivery and excellent outcomes.
